# Renaming of Hallervorden–Spatz disease: the second man behind the name of the disease

**DOI:** 10.1007/s00702-021-02408-x

**Published:** 2021-10-16

**Authors:** Luca Voges, Andreas Kupsch

**Affiliations:** 1 Department of Anesthesiology, Unfallkrankenhaus Berlin, Berlin, Germany; 2 Department of Neurology and Stereotactic Neurosurgery, Otto Von Guericke University Magdeburg, Magdeburg, Germany; 3 Neurology Moves, Academic Practice, Bismarckstr. 45-47, 10627 Berlin, Germany

**Keywords:** NBIA, Hallervorden-Spatz, PKAN, Euthanasia

## Abstract

Hallervorden–Spatz disease (HSD) has been recently renamed to pantothenate kinase-associated neurodegeneration (PKAN) and neurodegeneration with brain iron accumulation (NBIA), mainly due to the unethical behavior of Julius Hallervorden in the National Socialist (NS) euthanasia program of the Nazi Third Reich. The role of the second name giver in the NS euthanasia program is less clear. Hugo Spatz was the director of the Kaiser Wilhelm Institute for Brain Research in Berlin-Buch during World War II (WWII), renamed to Max Planck Institute after 1945. After the war, he headed the Max Planck Institute for Brain Research in Frankfurt am Main. The present study investigates the potential involvement of Hugo Spatz in the NS euthanasia program. In the present study, we compared a list of euthanasia victims from the German Federal Archive Berlin (30.146 cases published after the reunification of Germany, BArch R179) with the files of the collection of specimens from 1940 until 1945 of Hugo Spatz as listed in the Archive of the Max Planck Society Berlin-Dahlem (*n* = 305). Furthermore, the old term HSD and the new terms PKAN and NBIA were systematically searched in PubMed from 1946, through January 2019 to evaluate the renaming process from HSD to PKAN/NBIA. Following Hugo Spatz’s death in 1969 growing evidence indicated that he may have taken part in the NS euthanasia program. This study identifies 4 euthanized victims in the patient files of Hugo Spatz from 1940 to 1945, suggesting involvement of Hugo Spatz in the NS euthanasia program. This further strengthens the argument that the former HSD should be exclusively referred to as PKAN or NBIA.

**Supplementary Information:**

The online version contains supplementary material available at 10.1007/s00702-021-02408-x.

## Introduction

**Fig. 1:**
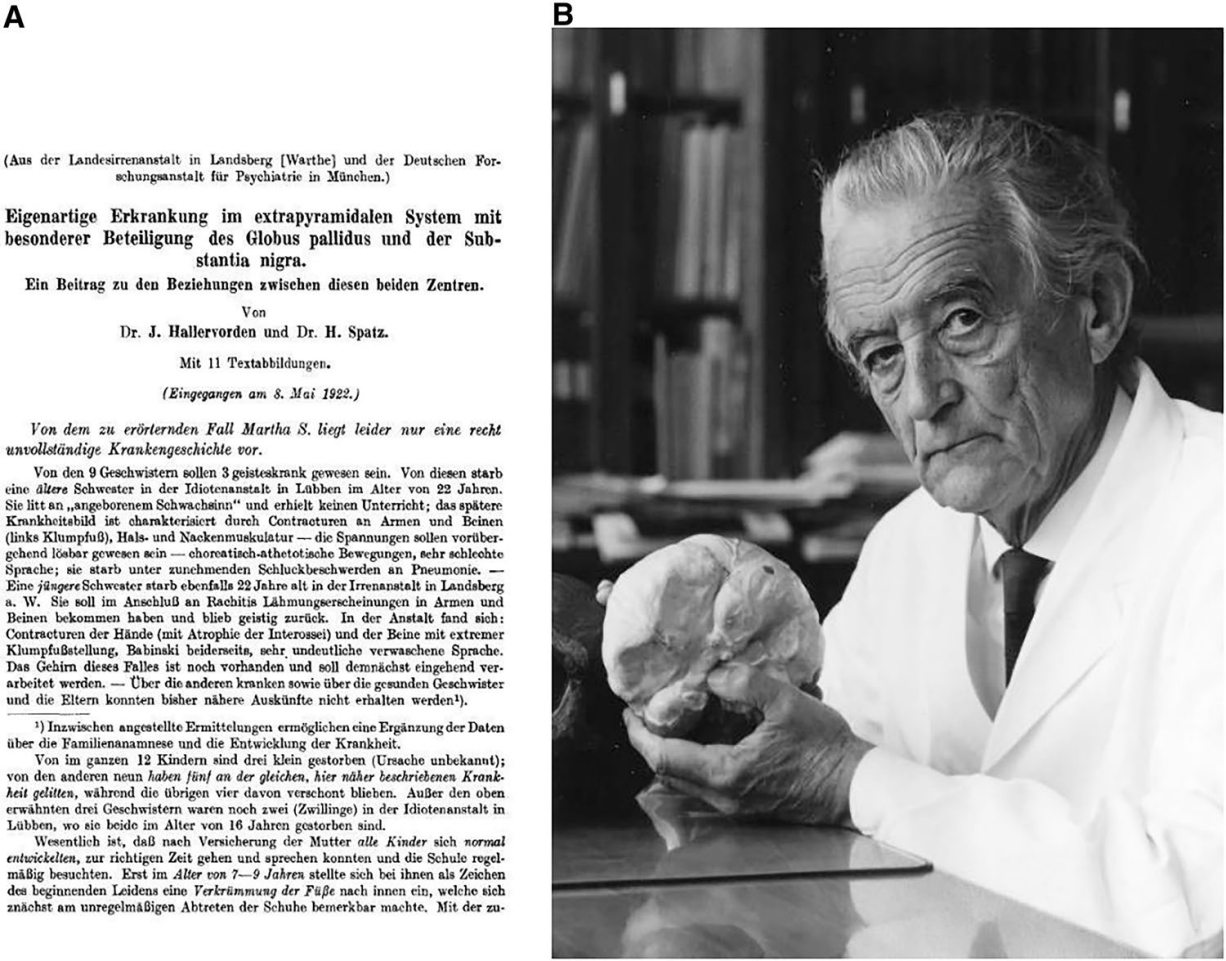
**a** First page of the publication of Spatz and Hallervorden, describing the disease NBIA. **b** Photography of late Hugo Spatz. *(Photos published with the permission of the Archive of the Max-Planck-Gesellschaft, Berlin-Dahlem.)*

In 1992 and 1996 Shevell (1992) and Harper (1996) first described the role of Julius Hallervorden and also Hugo Spatz (cf. supplement “H. Spatz’s Biography”) in a preliminary manner during the Nazi regime, suggesting that the disease should be renamed for ethical reasons (cf. Fig. 1 for German publication on neurodegeneration with brain iron accumulation (NBIA) and photography of Hugo Spatz). The two articles represent milestones as subsequently the term Hallervorden–Spatz disease (HSD) was largely replaced by pantothenate kinase-associated neurodegeneration (PKAN) and NBIA (Shevell 2012). However, the question remains: should a disease originally named for two equal discoverers, having been renamed due to the past unethical behavior of one, not be renamed for the sole presumed ethical discoverer?

The role of Julius Hallervorden has been repeatedly described and studied previously (Hughes 2007; Miller 2012; Shevell and Peiffer 2001; Wässle 2017; Zeidman 2011). The role of Hugo Spatz remains unclear, as already alluded to by Harper: “The direct role of Spatz is less clear; perhaps as director of the institute he did not choose to inquire closely into the details of a specific department’s work.”(Harper 1996). We looked at the potential involvement of Hugo Spatz in euthanasia activities during World War II (WWII). In this context, it should be noted that the German Neurological Society (DGN) named a well-known prize in honor of Hugo Spatz, which was changed to Adolf Wallenberg price in 1998. The present investigation moreover aims to investigate the success of the renaming process of HSD to PKAN and NBIA between 1946 and 2019.

## Methods

We used the following sources for the question of potential involvement of Hugo Spatz in the NS euthanasia program during the period 1937 to 1945:

German Federal Archive Berlin, with a list of 30.146 euthanasia victims (https://www.bundesarchiv.de/DE/Content/Artikel/Ueber-uns/Aus-unserer-Arbeit/euthanasie-im-dritten-reich.html) published after the reunification of Germany. These 30.146 patient files were found in the archive of the Ministry for State Security of the former German Democratic Republic, representing nearly half of the estimated 70.273 euthanasia victims of the so called “Aktion T4” (Aly 2012). How these 30.147 patient files found their way into the archive of the Ministry for State Security can only partly be reconstructed. Missing files were probably systematically destroyed before the end of WWII (Sandner 1999). The list of 30.146 euthanasia victims was compared to the files of the collection of brain specimens from 1940 to 1945 attributed to Hugo Spatz as listed in the Archive of the Max Planck Society Berlin-Dahlem (*n* = 305).

Matching was based on a manual comparison of available names (*n* = 305) between the Archive of the Max Planck Society Berlin-Dahlem and the list of 30.146 euthanasia victims from the German Federal Archive Berlin.

The German Federal Archive in Berlin was also consulted for information on activities of Hugo Spatz and Julius Hallervorden during the period 1939 until 1945 (cited as register numbers e.g. BArch).

Secondly, we evaluated the renaming process from HSD to PKAN/NBIA by searching PubMed from 1946 till January 2019 with the keywords “Hallervorden Spatz” and “PKAN/NBIA”. Publications that used the name HSD only and that used it in addition to PKAN/NBIA (for example indicated by “and”) were counted as publications using HSD, versus publications not using HSD.

## Results

We found 305 autopsy index cards in the Archive of the Max Planck Society Berlin matching with 4 of the 30.146 patient files in the German Federal Archive Berlin.

The following four victims of the NS euthanasia program have been identified, from the Hugo Spatz brain specimens in the Max Planck Society Archive.

**Fig. 2:**
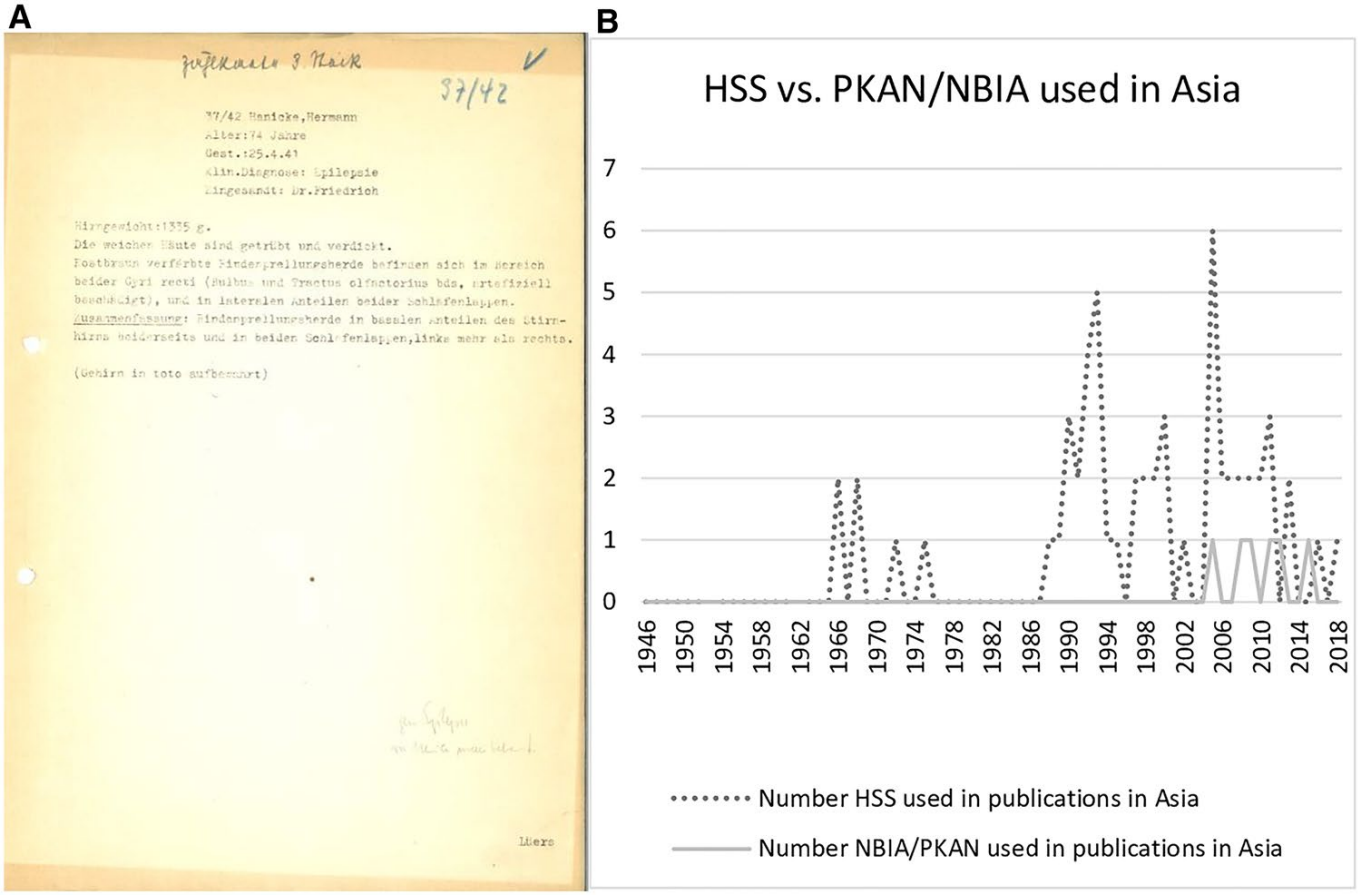
**a** Description of a brain specimen (file 37/42) from the collection of Hugo Spatz from 1941 at the Archive of the Max-Planck-Institute (present address: Boltzmannstrasse 14, 14195 Berlin, Germany). *Translation of File 37/42, highlighted in italics: 37/42 Hanicke Hermann, Age: 74, Died: 25.4.41, Clinical diagnosis: Epilepsy, send in by Dr. Friedrich, Brain weight: 1335 g. The soft meninges are dulled and thickened. Rubiginose coloured cortical contusions are situated in the area of both gyri recti (bulbus, tractus olfactorius on both sides) and in both lateral parts of temporal lobes. Summary: Cortical contusions in basal parts of the forehead brain on both sides and in both temporal lobes, left side more than right side. (brain completely stored).* **b** Diagram illustrating the number of HSS and NBIA/PKAN used in Asian publications from 1946 until 01-2019

E. A., born on 17.03.1885, diagnosed with epilepsy, deceased on 20.08.1940 [BArch(R179/2743; Hugo Spatz(III/54 Spatz, Hugo; Sig. 46; 3642).]; H. H., born 18.07.1867, diagnosed with epilepsy, deceased on 25.04.1941 [BArch(R179/12932); Hugo Spatz(III/54 Spatz, Hugo; Sig. 46; 3742)] (cf. Fig. 2A); W. P., born on 17.01.1916, diagnosed with epilepsy, deceased on 18.08.1941 [BArch(R179/6034); Hugo Spatz(III/54 Spatz, Hugo; Sig. 45; 3542)]; and P. S., born on 01.04.1875, diagnosed with epilepsy, deceased on 23.05.1941 [BArch(R179/12348); Hugo Spatz(III/54 Spatz, Hugo; Sig. 43; 3242)].

The second finding of the present study is that the Hallervorden–Spatz eponym was more often used in Asian literature during the last decade than in European and American literature. Between 1946 and 2019, 32 of 62 European and North American articles used ‘HSD’ (51.6%) and 26 of 35 Asian papers (74.2%) used the eponym.

## Discussion

The present study identifies four autopsy cases in the Archive of the Max Planck Society of Hugo Spatz as euthanasia victims. The names of these four patients were identified in a euthanasia victim list, published after the reunification of Germany in 1990 (Sandner 1999).

The present study cannot clearly identify whether Spatz requested or simply received autopsy material. So far there are only indications without clear evidence that Hugo Spatz requested brains of certain clinical manifestations complying with his scientific focus (Peiffer 2005, 2006; Schmuhl 2001, 2016).

Notably, the date of the autopsy and the date of the arrival of the autopsy material at the Kaiser-Wilhelm Institute are neither noted in the German Federal Archive nor in the Archive of the Max Planck Society.

Curiously, all four specimens in the present study suffered from epilepsy, which conflicts with Hugo Spatz’s major areas of interest during that period of time, i.e. traumatic brain injuries (Bak et al. 1971). It is unclear, how these specimens were included in the Spatz collection (ongoing research, personal communication January 2021, Prof. Dr. H. Czech, Institute for Medical History, Charité Berlin). Specimens could have been obtained by Spatz himself, collaborators or via other avenues.

What is clear, however, is that all four patients were killed during the “Aktion T4”, the centrally organized euthanasia program committed by the Third Reich from September 1939 until August 1941. During that stage of the NS euthanasia program, approximately 70.273 victims were killed in Germany (Aly 2012), not just for eugenic reasons but also for long term health cost savings (Peiffer 2005). After 1941 the NS euthanasia program still existed until the end of WWII in a decentralized manner leading to the extermination of another estimated 120.000 victims (Müller-Hill 1998).

Importantly, one previous study (Peiffer 1999, 2000) claimed distinctively higher numbers of euthanasia victims in the autopsy collection of Hugo Spatz.

The main methodological difference between the Peiffer study and the present study relates to Peiffer’s development of a list with seven criteria to define deceased euthanasia victims. If one of seven criteria applied to the death of a person, they were deemed to be a victim of euthanasia.

The seven criteria defined by Peiffer are the following (Peiffer 2000, p.159):

1. Detection of Z or Be-numbers or other registration numbers of the death camps
2. Brains of patients, whose index cards have a notation of a death camp on them (for example “D”)
3. Documentation of the “Reich Committee for the Scientific Registration of Serious Hereditary and Congenital Illnesses”
4. Names on transport lists of death camps
5. Brains obtained during the active time from death camps
6. Brains from centers, in which the physicians worked under an obligation of confidentiality or received additional money from the “T4” headquarters
7. Brains of patients with the notation “transferred to an unknown institution” or “transferred by the order of the Reich Defense Commissioner” in their medical history

In contrast, the present study employed a manual comparison of a list of 30.146 euthanasia victims in the Federal Archive Berlin with the files of the specimen collection of Hugo Spatz from 1940 until 1945 (Archive of the Max Planck Society Berlin-Dahlem), explaining the lower numbers in the present study.

Likely, Hugo Spatz knew about the involvement of his friend and colleague, Julius Hallervorden, in the NS euthanasia program. Specifically, Hugo Spatz participated in a conference of the board of the Kaiser Wilhelm Institute in 1938, in which the board discussed the possibilities of investigating brains of killed patients from the pediatric clinic of Görden, Brandenburg, located between Magdeburg and Potsdam (BArch R4901/14104, pp. 5–14, 70–77). Future research may help to elucidate Hugo Spatz’s role in this conference, but is beyond the scope of the present study.

Notably, Julius Hallervorden was the leading pathologist at the pediatric clinic in Görden from 1929 till 1945 (Seidelman 2012; Schmuhl 2000) and was present when 60 children were killed at the pediatric clinic of Görden, Brandenburg on October 28th, 1940 (Beddies 2009; Schmuhl 2000). Compared to other medical scientists who specifically requested brains or killed patients themselves (for example Arthur Schreck who was the deputy director of the hospital “Heil und Pflegeanstalt” Wiesloch), Julius Hallervorden seemed to draw an opportunistic exploitation of the so called “Aktion T4” (Middelhoff 1993; Miller 2012). In addition, Hugo Spatz wrote a letter to Maximinian de Crinis to obtain funding for Julius Hallervorden to use these brains for his scientific research on “idiocy in childhood” (BArch R73/14825, p. 3125).

The contact of Hugo Spatz with NS medical activities included his interactions with leading NS doctors involved in euthanasia (e.g. Hans Heinze, Maximinian de Crinis (Nedoschill and Castell 2001; Jasper 1991, BArch R4901/14104, pp. 5–14; 70–77), which of course does not reflect nefarious activities, but may rather mirror the banality of evil (Arendt 1963).

After WWII, Hugo Spatz denied any involvement in the use of brains from euthanasia victims for research at the Kaiser Wilhelm Institute for Brain Research during interrogations by U.S. American investigators (Topp 2013). Thus, in 1945 the Jewish Austrian doctor Major Leo Alexander independently questioned Julius Hallervorden and Hugo Spatz about the potential connection between their work and the euthanasia program with conflicting results (Alexander 1949). While Julius Hallervorden admitted that the Kaiser Wilhelm Institute for brain research consciously used brains of euthanasia victims for their research, Hugo Spatz explicitly denied any relations between his institute and the euthanasia program (Zeidman and Pandey 2012).

Certainly, more research is warranted to further assess potentially criminal and unethical involvement of Hugo Spatz. Our independent findings of four isolated cases add further weight to renaming HSD to PKAN.

Furthermore, our research showed a declining use of the Hallervorden–Spatz eponym in medical literature (Shevell 2012), although still used today, particularly in Asian countries (cf. Fig. 2B). For instance, from 2010 to 2018, eleven publications in Asia used HSD to refer to PKAN/NBIA. One possible explanation may relate to lower awareness in Asian countries of the history of euthanasia during the Third Reich.

Importantly, the prestigious Hugo Spatz Award was granted until 1998 to honour scientific achievements on behalf of the official German Society of Neurology (DGN). A distinct debate among German leading neurologists almost prevented the renaming of the Hugo Spatz Award to the Adolf Wallenberg Award (Back 2020), showing that renaming of disease entities (Kondziella 2009) should also apply to awards.

Adolf Wallenberg (1862–1949), was a German Jewish doctor most known for describing Wallenberg syndrome, a brain stem syndrome comprising ipsi- and contralateral neurological symptoms (Zeidman and Mohan 2014).

## Conclusion

We identified four euthanasia victims found in the collection of Spatz specimens (archive of the MPI). Due to the lack of evidence for specific requests of Hugo Spatz to obtain euthanasia material and of scientific reports on the four euthanasia victims, the possibility remains that the four brains were examined by a third party and only subsumed to the Hugo Spatz collection during the evacuation of Berlin-Buch 1945. However, the present results show that the process of investigating the political complicity of medical practitioners and researchers is far from being complete in Germany. This is also reflected by the opposing reaction of the DGN in renaming the former Hugo Spatz Award to the Adolf Wallenberg Award in 1998.

## Supplementary Information

Below is the link to the electronic supplementary material.

Supplementary file1 (DOCX 44 KB)

## Abbreviations

BArch: Federal Archive of Germany (Bundesarchiv)
DGN: Deutsche Gesellschaft für Neurologie
HSD: Hallervorden–Spatz disease
NBIA: Neurodegeneration with brain iron accumulation
NS: National Socialist
PKAN: Pantothenate kinase-associated neurodegeneration
PubMed: Medical online data base
WWII: World War II
