# Supplementary material for: Renaming of Hallervorden–Spatz disease: the second man behind the name of the disease
Source: J Neural Transm (Vienna). 2021 Oct 16;128(11):1635–40. doi: 10.1007/s00702-021-02408-x (PMC8536572; doi:10.1007/s00702-021-02408-x)
Supplement: Supplementary file 1 — Supplementary file1 (DOCX 44 KB) [file 702_2021_2408_MOESM1_ESM.docx]

**Supplement**

**H. SPATZ’S BIOGRAPHY**

Hugo Spatz was born on September 2, 1888 as son of Bernhard Spatz and Julie Spatz, born Heinzelmann in Munich [1]. His father was a general practitioner and editor of the Munich medical weekly journal (Münchner Medizinische Wochenschrift) [2,3]. Hugo Spatz visited elementary school from 1894 until 1898 in Munich and subsequently attended the German high school (Theresien-Gymnasium) in Munich, where he finished school with the German university-entrance diploma (Abitur) [4].

Hugo Spatz began his medical studies in Munich, where he passed the preclinical exam (Physikum) in 1910 [3,4]. He continued his studies in Heidelberg, where he was acquainted with the well-known psychiatrist and scientist Franz Nissl who supervised his dissertation with the title “Histology and development of the rabbit spinal cord”, which was finished in July 1914 [5]. During his studies as a medical assistant in the histological laboratory of the psychiatric clinic under supervision of Franz Nissl he was first introduced into scientific research of the central nervous system [5,6]. In 1912 Hugo Spatz passed the second medical state exam in Heidelberg. He continued his clinical career as a sub-intern at the psychiatric clinic in Heidelberg (Universitäre Irrenklinik) and the German hospital in Istanbul [4]. In January 1914, he was approved as German doctor (Approbation) [7]. From March 1^st^ 1914, until the beginning of World War I in August 1914, following the assassination of the Austrian heir apparent, Hugo Spatz served as voluntary assistant at the University of Heidelberg [4].

During WWI Hugo Spatz served as assistant and consultant doctor in position battles at Verdun, Reims and Messines [8]. Following the end of WW I in November 1918 he followed his academic teacher Franz Nissl, who had become leader of the histopathological department of the German research Institute for Psychiatry in Munich under the supervision of Emil Kraeplin and was employed as research assistant starting on January 1^st^ ,1919 [3].

Following Nissl’s death in 1919 Hugo Spatz moved to Freiburg and worked as Scientific Assistant at the Anatomic Institute under the supervision of Eugen Fischer. Subsequently he returned to Munich and continued to work as Scientific Assistant under the supervision of Walter Spielmeyer [6]. Here he first met and started to collaborate with Julius Hallervorden, who worked as a visiting scientist at the same institute and with whom he collaborated closely. In 1922 they described together the case of a young girl Martha S., which led to the discovery of the disease NBIA (formerly Hallervorden-Spatz Syndrome) [9].

In 04.08.1923 Spatz habilitated (German venia legendi) in Psychiatry and Neurology. In April 1924 Hugo Spatz became full assistant at the psychiatric and genetic clinic Munich. Two years later Emil Kraeplin died, and Oswald Bumke became the new director of the German Institute for Psychiatry in Munich [3,4]. The relationship between Oswald Bumke and Hugo Spatz was characterized by a distant and professional contact. Despite the spatial separation Hugo Spatz kept up the good relationship with Walter Spielmeyer. The relationship to Oswald Bumke remained distant [6].

Four years later (1930) Spatz became consultant doctor and leader of the Anatomic laboratory at the psychiatric clinic in Munich. On August 4^th^, 1923 he became assistant professor for psychiatry at the University of Munich (Privatdozent) and on July 28^th^, 1927 he was promoted to a full professor (German “amtsbezogen”) [3,4].


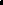


He became scientific member of the Kaiser-Wilhelm Society for advancement of sciences. He was awarded for his works with the Acchucarro Price for Neurology in Madrid. Furthermore, he became corresponding member of the Society of German Doctors in Prague on October 1931 [10,11]. On 18.05.1933, being 45 years old, he married Ortrud Möllendorf, daughter of his boyhood friend Wilhelm von Möllendorf [12]. In April 1937 Hugo Spatz succeeded Oscar Vogt as director of the Kaiser-Wilhelm Institute for brain research in Berlin-Buch and at the same time became leader of the neuroanatomic department at the same institution, declining to become successor of Walter Spielmeyer as director of the histological department of the German Research Institute in Munich. Under the leadership of Hugo Spatz the KWI was intensively restructured with a close cooperation of the other clinical departments in Berlin. Specifically, he presented his friend and colleague Julius Hallervorden, as leader of the newly founded department for histopathology. Hallervorden was at the same time leader of the department of pathology of the psychiatric hospitals of Brandenburg in Potsdam which subsequently moved to Brandenburg-Görden in 1938 [13]. On the March 1^st^, 1937 Spatz joined the Nazi party [14]. At the beginning of WWII Hugo Spatz was appointed on August 26^th^, 1939 as “Oberfeldarzt” and importantly the Institute became part of the German army, which lead to the foundation of three new departments: 1.)The institute for Advanced Research for lesions of the central nervous system of the medical military academy under the supervision of Julius Hallervorden and the military leadership of Bernhard Patzig, who worked already under Oscar Vogt at the KWI for brain research (leader of the institute for human genetics). 2.) A department for brain research of the aviation medical research institute of the German Air Force under the supervision of Hugo Spatz. 3.) A research department for brain, spinal and peripheral nerve injuries under the leadership of Wilhelm Tönnis [13]. The research of the newly founded institute of Hugo Spatz comprised open and closed lesions of the brain. According to the Alexander Report (Major Leo Alexander was authorized by the Supreme Headquaters Allied Expeditionary Forces to examine the work of German medical research institutes, and he wrote several reports about his results), Hugo Spatz obtained brains from all fallen members of the German Air Force in the external division of the aviation medical research institute [15].

The research institute was converted into a hospital for brain injured members of the German army. During the army attacks (airport attacks) in winter 1944/1945 the institute in Berlin was dissolved and moved to different places: Dillenburg, Göttingen and Schleswig. In march 26th 1945 Spatz left Berlin and moved part of his collections to the new neuroanatomic department in Munich [6,13]. Following the end of the war Spatz was arrested on July 20^th^, 1945 because of his leading position and transferred to a military camp in Garmisch-Partenkirchen on July 31th, 1945 [16]. Here Spatz was asked for his activities during war, and the American authorities tried to detect the state of the art of the German research in neuroscience. They concluded that Hugo Spatz only served as a pure scientist during the war and did not show any political interest [16,17, files can be provided upon request]. Following his release in June 1946, Spatz worked as scientific advisor and assistant at the American Aeromedical Center, writing about his experiences with injuries of the central nervous system in aircraft, which cumulated in the only English publication “brain injuries in aviation” by HS [18]. In 1947 HS followed his friend and colleague Julius Hallervorden to Dillenburg, where Hallervorden had collected material of the KWI for research. Subsequently Spatz was able to continue his work despite limited facilities [13]. In 1948 the Max-Planck Society succeeded the KWI and in 1949 the institute, now officially called Max-Planck Institute for brain research, moved to Gießen. Spatz was able to recruit sufficient research facilities for his departments to continue his work [19]. Hugo Spatz subsequently led the Max-Planck Institute for brain research from 1948 until he became an emeritus professor in 1959 [6]. After his retirement, he continued to lead the research group until the institute moved to Frankfurt in 1962. On 27.01.1969 Spatz died after short disease in Frankfurt, Main [6,13].

**References**

1. BArch (R4901) Karteikarte Reichärztekammer, Spatz, Hugo

2. Janzen R. (1976) Laudatio for Hugo Spatz. MMW Munch Med Wochenschr. 118:511-2

3. BArch (VBS 1/1110068741), p. 974

4. BArch (VBS 307/ 8200002962), p. 2066

5. Hassler R. (1969) In memoriam Hugo Spatz, September 2, 1888-January 27: Dtsch Z Nervenheilkd., Band 195: ii256

6. Bak I, Christ J, Hassler R, Hossmann K, Kahle W, Krücke W, Peters G, Stephen H, Tönnis W, Zülch K (1971) Leben und Werk von Hugo Spatz. In: Max-Planck- Institut für Hirnforschung (Hrsg) Dokumentationsstelle MPG Göttingen, Germany

7. BArch (VBS 1/1110068741), p. 938

8. BArch (VBS 307/ 8200002962), p. 2068

9. Hallervorden J, Spatz H. (1922) Eigenartige Erkrankung im extrapyramidalen System mit besonderer Beteiligung des Globus Pallidus und der Substantia Nigra: Z. f. d. g. Neur. u. Psych., 79:254–302

10. Lindenberg R. (1970) In memoriam; Dr. Hugo Spatz. J Neuropathol Exp Neurol. ,29:331-4

11. Scholz W. (1969) Hugo Spatz 1888-1969. Arch Psychiatr Nervenkr., 212:91-96

12. Stephan H. (1969) Hugo Spatz 1888-1969. J Hirnforsch. 11:199-202

13. Schmuhl HW (2000) Hirnforschung und Krankenmord. Das Kaiser-Wilhelm-Institut für Hirnforschung 1937 – 1945. Vorabdrucke aus dem Forschungsprogramm „Geschichte der Kaiser-Wilhelm-Gesellschaft im Nationalsozialismus". In: Carola Sachse (Hrsg) Präsidentenkomission der Max-Planck-Gesellschaft, Berlin, Germany, pp 1–62

14. BArch (R4901), parteistaatlicher Erhebungsbogen, Spatz, Hugo

15. Alexander L (1949) Medical science under dictatorship. N. Eng J. Med 241: 39–47

16. Records of the Army Staff (Record Group 319) IRR-Personal Name File selected Printouts of digital Intelligence and investigative dossiers: Spatz, Hugo  - D191922 & XE191922: 58  pages (files in RM 2400 -Cookson).

17. Department of the Army. Office of the Assistant Chief of Staff, G-2, Intelligence. Collections and Dissemination Division. Document Library Branch. (1950 - 1955)
Series : Publications Files, compiled 1950 - 1951, NM3 82 Box 677 @ 270: 8/13/5 -Item: Clos Report: XXVII-96, 24 - Oberfeldarzt Professor Hugo Spatz, The Department of Brain Research, Kaiser Wilhelm Institute: 12 pages.

18. Spatz H. (1950) Brain injuries in aviation in: German Aviation Medicine World War II, Dept. of the air force 1:616–640

19. Topp S, Peiffer J (2007) Das MPI für Hirnforschung in Gießen: Institutskrise nach 1945, die Hypothek der NS-„Euthanasie“ und das Schweigen der Fakultät. In: Oehler-Klein (Hrsg) Die Medizinische Fakultät der Universität Gießen im Nationalsozialismus und in der Nachkriegszeit: Personen und Institutionen, Umbrüche und Kontinuitäten, Stuttgart, Germany
